# A Narrative Review on Cognitive Impairment in Type 2 Diabetes: Global Trends and Diagnostic Approaches

**DOI:** 10.3390/biomedicines13020473

**Published:** 2025-02-14

**Authors:** Xiaobin Liao, Yibin Zhang, Jialu Xu, Jiaxin Yin, Shan Li, Kun Dong, Xiaoli Shi, Weijie Xu, Delin Ma, Xi Chen, Xuefeng Yu, Yan Yang

**Affiliations:** 1 Department of Endocrinology, Tongji Hospital, Tongji Medical College, Huazhong University of Science and Technology, Wuhan 430030, China; 19977295795@163.com (X.L.); 17343975167@163.com (Y.Z.); jlxu93@163.com (J.X.); yinjiaxin6892@163.com (J.Y.); lishandoctor@163.com (S.L.); kundong2009@aliyun.com (K.D.); fll766@163.com (X.S.); xwj.07@163.com (W.X.); maderine4@163.com (D.M.); amazingamycx@163.com (X.C.); xfyu188@163.com (X.Y.); 2 Second Clinical College, Tongji Medical College, Huazhong University of Science and Technology, Wuhan 430030, China; 3 Branch of National Clinical Research Center for Metabolic Diseases, Wuhan 430030, China

**Keywords:** diabetes mellitus, cognitive dysfunction, biomarkers, diagnosis

## Abstract

Diabetes is a chronic disease that affects many people, with both its incidence and prevalence rising globally. Diabetes can lead to various complications, among which cognitive impairment in diabetic patients significantly impacts their daily life and blood glucose management, complicating treatment and worsening prognosis. Therefore, the early diagnosis and treatment of cognitive impairment are essential to ensure the health of diabetic patients. However, there is currently no widely accepted and effective method for the early diagnosis of diabetes-related cognitive impairment. This review aims to summarize potential screening and diagnostic methods, as well as biomarkers, for cognitive impairment in diabetes, including retinal structure and function examination, brain imaging, and peripheral blood biomarkers, providing valuable information and support for clinical decision making and future research.

## 1. Introduction

Diabetes mellitus (DM) is a chronic disease characterized by hyperglycemia, which is caused by the absolutely or relatively insufficient secretion and utilization of insulin. The prevalence of diabetes mellitus is increasing worldwide. The global age-standardized prevalence of diabetes increased by 90.5% (95%UI 85.8–93.6) between 1990 and 2021, from 3.2% (3.0–3.5) to 6.1%. There were 529 million people living with diabetes globally in 2021, and this number is expected to more than double to approximately 1.31 billion by 2050 [1]. In recent years, diabetes-related cognitive impairment has gradually become one of the common chronic complications of diabetes. In adults with type 2 diabetes mellitus (T2DM), cognitive deficits can be categorized into three stages based on severity: diabetes-related cognitive decline, mild cognitive impairment (MCI), and dementia [2]. MCI refers to a progressive decline in memory or other cognitive functions without impairing the ability to perform activities of daily living. It is generally considered to be an intermediate stage between the expected cognitive decline due to normal aging and dementia. There are many differences in cognitive impairment (CI) between T2DM and non-diabetic individuals. Cognitive decline in patients with T2DM is often manifested in memory, executive function, and information processing speed [3]. CI in non-diabetic people is mainly caused by memory impairment, and cognitive function usually declines faster. At present, the mechanism of CI caused by diabetes is still under study. A variety of potential mechanisms have been shown to have an important role in diabetes-related cognitive impairment, such as insulin resistance, neuroinflammation, chronic hyperglycemia, and oxidative stress.

Epidemiological data show that diabetes is an important risk factor for CI. A cohort study of 1891 individuals found that patients with T2DM were significantly more likely to develop CI (odds ratio = 1.86, 95% CI: 1.39–2.49) [4]. In addition, diabetes can accelerate the development of CI. A meta-analysis of 144 prospective studies showed that diabetes leads to a 1.25- to 1.91-fold increased risk for cognitive disorders (CI and dementia) [5]. A 2021 study showed that poor diabetes control was associated with a doubling of the risk for CI and a threefold increase in the risk for progression from CI to dementia [6].

Cognitive impairment secondary to diabetes can have a negative impact on patients’ daily lives, leading to increased needs for personal care, higher hospitalization rates, and a greater likelihood of developing depression [7]. This not only hinders patients’ ability to care for themselves but also results in higher healthcare costs, creating an economic burden on both families and society. Additionally, blood sugar control in patients with diabetes and CI is often problematic. Both hyperglycemia and hypoglycemia can adversely affect cognitive function. Studies have shown that higher average blood glucose levels in diabetic patients are associated with an increased risk of dementia (*p* = 0.002). At a glucose level of 10.5 mmol/L versus 8.9 mmol/L, the adjusted hazard ratio for dementia was found to be 1.40 (95% CI, 1.12 to 1.76) [8]. During acute hypoglycemia, diabetic patients showed significant declines in performance on short-term memory, delayed memory, and working memory tests, with working memory and delayed memory being most severely affected [9]. Poor blood glucose control appears to be a risk factor for cognitive decline, which, in turn, exacerbates the severity of CI in diabetic patients, creating a vicious cycle that undoubtedly complicates their treatment and care. Furthermore, multiple studies on individuals with impaired glucose tolerance but without diabetes have shown that, compared to the control group, they scored lower on the Mini-Mental State Examination (MMSE), had poorer long-term memory, reduced verbal fluency, and exhibited a higher incidence of Alzheimer’s disease (AD) and vascular dementia [7]. This suggests that some diabetic patients may experience cognitive decline even in the early or preclinical stages of diabetes. Therefore, the early diagnosis and treatment of T2DM patients with CI are beneficial to delay cognitive decline and improve prognosis. Although there are currently many methods for detecting CI, there remains controversy regarding their effectiveness, particularly in the early diagnosis of diabetes-related cognitive impairment. The American Diabetes Association (ADA) guidelines from 2020 recommend simple cognitive screening tools, such as the MMSE, the Montreal Cognitive Assessment (MoCA), and Mini-Cog, for assessing cognitive function in diabetic patients [10]. However, these screening methods seem to be influenced by other factors and have relatively low sensitivity. Therefore, this review aims to summarize and evaluate potential screening and diagnostic methods, as well as biomarkers for CI in diabetes. The goal is to provide information for clinical decision making and future research, with the aim of identifying valuable, clinically applicable diagnostic tools for early detection and intervention, ultimately improving patient outcomes.

## 2. Screening Methods for Diabetic Cognitive Impairment Recommended by ADA Guidelines

The ADA recommends early MCI screening in patients with T2DM aged 65 years and older. They recommended the use of some simple assessment tools for screening CI, such as MMSE, MoCA, and Mini-Cog. This article focuses on the MMSE and MoCA, two commonly used screening tools [10].

### 2.1. MMSE

The MMSE is a 30-point questionnaire widely used in clinical and research studies to evaluate attention and orientation, memory, registration, recall, computation, language, and the ability to draw a complex polygon [11]. MMSE was less sensitive to the recognition of patients with MCI. One study compared the validity of the MMSE with various gold standards, and standard validity measures showed higher levels of sensitivity for moderate to severe CI and lower sensitivity for MCI [12]. This may be due to the presence of a ceiling effect. It means that most of MMSE’s test items are relatively simple, and only severe CI would prevent patients from answering most of the test items correctly. In addition, MMSE has many advantages and limitations, as shown in Table 1.

### 2.2. MoCA

The MoCA is a rapid screening tool for CI. The MoCA, like the MMSE, is a test with a total score of 30, with higher scores indicating better cognitive performance. The test included 11 items in 8 cognitive domains including attention and concentration, executive function, memory, language, visual structure skills, abstract thinking, calculation and orientation. A score of 26 is usually taken as the critical score for MoCA, and a score of 25 or less is indicative of MCI. MoCA is more effective than MMSE in differentiating mild cognitive defects from non-cognitive defects [13,14,15]. The superior sensitivity of MoCA in MCI detection may be explained by several features of its design. Compared to MMSE, MoCA’s memory test involved more words, fewer learning trials, and longer recall delays. MCI participants may also have mild impairments in executive functioning, higher-level language, and complex visuospatial processing. Compared with MMSE, MoCA can better distinguish the changes in the above parts by using more complex and demanding tasks [16].

Although both the MMSE and MoCA have limitations in identifying diabetes-related cognitive impairment, we may consider combining these two approaches with other diagnostic methods (e.g., biomarkers and imaging markers) to improve diagnostic sensitivity.

## 3. Retinal Structure and Function Examination

Studies have shown that people with T2DM and MCI exhibit structural and metabolic alterations in the retina, suggesting that noninvasive retinal markers may be useful for detecting T2DM patients at risk for cognitive dysfunction [17]. The retina originates from the neural tube and is part of the central nervous system during embryonic development [18]. Based on embryological knowledge, the retina can be viewed as an extension of the brain, and both organs show similar patterns of vascularization during development [19,20,21,22]. Since the retina has similar embryonic origin, anatomical characteristics and physiological characteristics to the brain, the existence of diabetic retinopathy (DR) may indicate the damage of the cerebrovascular system and brain nerves [23,24]. An association between DR and CI has been reported [23,24,25].

Advanced retinal imaging technology can help us identify retinal lesions at an early stage. Based on the association between DR and CI, retinal imaging may be a potential screening tool for the early diagnosis of CI. Optical coherence tomography (OCT) is a rapid, noninvasive imaging tool that produces 3D cross-sectional images of the retina and facilitates the precise and accurate measurement of the thickness of individual retinal components [26]. Spectral-domain OCT (SD-OCT) is a more sophisticated technique currently used in clinical settings. SD-OCT can image the retina in three dimensions with higher scan speed, higher axial resolution, and lower measurement variability [27]. The retinal nerve fiber layer (RNFL) is the innermost layer of the retina and is composed of retinal ganglion cell axons that connect the lateral side of the neural retina to the dorsal lateral geniculate nucleus, with synaptic connections leading to the visual cortex [28]. Thinning of the retinal nerve fiber layer measured through optical coherence tomography retina scans has been reported in individuals with MCI and AD [29,30]. Several systematic reviews and meta-analyses have also reported that patients with MCI and AD exhibit thinning of the RNFL [30,31,32]. In a community-based cohort study, an association between thinner RNFL and worse cognitive function was observed in individuals without neurodegenerative disease, indicating an increased likelihood of future cognitive decline [33]. Thus, the measurement of RNFL thickness using OCT imaging may play a role in screening those at risk for future cognitive decline.

Recent studies indicate that neurodegeneration in the retina may occur early in the development of DR [34]. Thus, the assessment of retinal parameters that are associated with neurodegeneration, such as retinal function, may help to identify patients with T2DM who have CI. Multifocal electroretinogram (mfERG) has proven to be an invaluable tool for studying neuroretinal function in DR. The mfERG is an electrophysiological examination. It is capable of stimulating multiple retinal regions simultaneously and recording each response independently, thereby providing a topographic measure of retinal electrophysiological activity. It can record many local cone-driven ERG signals under light-adapted conditions, thereby detecting small areas of retinal dysfunction in this region [35]. However, this method is complex, requires specialized personnel, and takes a long time to test, and is mainly used in clinical trials [36].

Microperimetry (MP), also known as fundus-driven perimetry, is a reliable tool for assessing retinal function [37]. MP is a straightforward, rapid, and noninvasive examination that quantifies retinal sensitivity by determining the minimum light intensity required for a patient to perceive a light spot stimulating a specific region of the retina, while also evaluating fixation stability [38]. Assessing retinal sensitivity and eye fixation through MP has been proposed as a non-invasive retinal tool for identifying and monitoring MCI in patients with T2DM [39,40]. Research indicates that retinal sensitivity assessed through MP is associated with topographic measures of brain tissue degeneration and reduced brain metabolism. Additionally, individuals with T2DM and MCI exhibit lower retinal sensitivity compared to those who are cognitively healthy [40]. MP equipment has been continuously developed in the last few decades, and a variety of MP devices have been used in clinical and research applications. Since different instruments and settings are used in different populations, inter-device differences should be considered. Studies shows that the retinal sensitivity reported by these devices is affected by the brightness, contrast sensitivity, and background lighting of the devices themselves [41]. In addition, MP also has limitations as a screening tool for CI. First, this device may not be widely used in many primary or even tertiary healthcare institutions [42]. Second, there appear to be no studies that have specifically assessed whether testing is influenced by psychological or emotional states [42]. Ultimately, the presence of advanced DR could serve as a constraint. However, we suggest utilizing it as a means for the early identification of cognitive decline. At this point, many individuals with T2DM exhibit only mild to moderate levels of DR [42]. Given the reasons mentioned, retinal microperimetry can still be viewed as a developing and potentially valuable method that needs additional validation and examination.

In Table 2, we have summarized three methods: OCT, mfERG, and MP.

## 4. Diabetes-Specific Dementia Risk Score

In 2013, the diabetes-specific dementia risk score (DSDRS) was developed to help researchers and clinicians identify individuals with T2DM who are at risk of developing dementia. The DSDRS estimates a person’s overall risk of developing dementia within the next decade based on diabetes-related co-morbidities and complications, age, and education level [43]. The DSDRS serves as an effective instrument. All the predictors considered are simple to evaluate and easily accessible in primary care environments. Notably, it does not require any additional costly or labor-intensive assessments like cognitive evaluations or brain scans. Among the predictors featured in the final model were complications related to end organs [43]. The DSDRS individual total score, which varies from −1 (indicating low risk) to 19 (indicating high risk), was determined by adding together the relative contributions of each predictor as outlined in the original model [44]. Since electronic medical records are used in most developed countries, we can obtain relevant data from the patient’s medical records (age, education level, cerebrovascular disease, cardiovascular disease, diabetic foot, etc.) to calculate the DSDRS score, which is an advantage of DSDRS. The DSDRS categorized individuals into 14 groups, with scores ranging from −1 to ≥12, demonstrating a 15-fold variation in dementia risk between the lowest and highest total scores [43]. The results of the Spanish MOPEAD (Models of Patient Engagement for Alzheimer’s Disease) study showed that the predictive value of DSDRS ≥ 7 as a screening tool for CI was AUROC (Area Under the Receiver Operating Characteristic curve) = 0.739, *p* = 0.024, CI 95% (0.609–0.825). When DSDRSs were combined with MMSE scores, the predictive value of CI was significantly improved (AUROC = 0.902, *p* = 0.003, CI 95% (0.840–0.992)) [45]. A study involving 2694 patients with T2DM found that a higher DSDRS score was associated with an increased risk of CI 2.5 years later, as well as more subtle declines in cognitive function over time [44]. These results suggest that the DSDRS may be a reliable screening tool for cognitive decline in T2DM patients > 65 years of age.

## 5. Brain Imaging

Diabetes causes structural and functional changes in the brain. Over the past few decades, brain imaging has proven to be an important means of studying brain changes in aging and age-related cognitive decline. The brain imaging changes in T2DM patients with CI include brain parenchymal damage, cerebrovascular disease, changes in brain function, and changes in brain metabolism. Over the last two decades, advanced brain imaging methods—including structural, functional, and diffusion magnetic resonance imaging (MRI), along with positron emission tomography (PET)—have proven effective in studying brain alterations in individuals with T2DM and MCI.

### 5.1. Brain Structural Changes

Gray matter (GM) and white matter (WM) are two sensitive markers in brain imaging studies that indicate brain structural changes in patients with T2DM. Voxel-based morphometry (VBM) and surface-based morphometry (SBM) are two crucial techniques used to investigate alterations in GM within the brain [46]. VBM is a well-established, fully automated quantitative MRI approach that is extensively utilized to describe neuropathological changes in the brain. Previous VBM studies have shown that both T2DM patients with and without MCI exhibit a significant reduction in total GM volume compared to healthy controls (HC), and the degree of GM volume loss is greater in MCI patients with T2DM than in patients without MCI [47]. In contrast to VBM, the surface-based approach offers a more precise assessment of GM volume at a subvoxel level [48]. An SBM study showed that patients with T2DM-MCI exhibited a relatively extensive pattern of cortical GM loss involving temporal, occipital, parietal, and cingulate cortical regions [49].

Diffusion tensor imaging (DTI) is a crucial technique for investigating alterations in WM associated with T2DM. DTI measures both the directionality and extent of the three-dimensional random diffusion of water molecules within soft tissues. The primary indicators used in DTI are fractional anisotropy (FA) and mean diffusivity (MD). FA indicates the level of anisotropic movement of water molecules, whereas MD assesses the average motion of these molecules in the three possible dimensional directions [50]. DTI enables the assessment of overall alterations in WM, as well as the identification of specific tract abnormalities and the integrity of brain networks [51,52]. Research using DTI in individuals with T2DM has demonstrated alterations in WM microstructure and connectivity relative to control groups, which are linked to CI [53]. In conclusion, GM and WM abnormalities play a crucial role in the development of CI in diabetes.

### 5.2. Alterations in Cerebral Metabolism

Noninvasive neuroimaging techniques have played an important role in identifying metabolic biomarkers in the brain. Among these, PET utilizing fluorine-18 (^18^F)-labeled 2-fluoro-2-deoxy-d-glucose (^18^FDG) as a tracer and proton magnetic resonance spectroscopy (^1^H MRS) are recognized as reliable techniques [54]. PET is a quantitative imaging method that utilizes radionuclides, relying on the detection of a radiolabeled positron-emitting tracer injected peripherally as it travels through or gathers in the brain [52]. ^18^FDG is a tracer for studying brain glucose metabolism. It can be transported to the brain in a similar manner to glucose, but unlike glucose, it cannot be further metabolized after being phosphorylated to FDG-6-P, thus accumulating in brain tissue. Decreases in the regional cerebral glucose metabolic rate, assessed through FDG-PET, are linked to a higher risk of AD and can be detected several years prior to the onset of dementia [55].

MRS exploits the spin properties of certain nuclei upon entry into a magnetic field, and these spin properties are used to determine the concentration of specific metabolites in the tissues examined [56]. ^1^H-MRS has been used to identify and characterize metabolic changes associated with many neurological diseases. Metabolites detectable by ^1^H-MRS include N-acetylaspartate (NAA), choline (Cho), creatine (Cr), and others. NAA is a marker of neurons and reflects the number and functional status of neurons. Cho plays a role in the composition of cell membranes and the formation of myelin. Cr is linked to energy metabolism [57]. An analysis of ^1^H MRS Data from diabetic patients revealed significant alterations in the levels of NAA, Cr, Cho, myo-inositol, glutamate, and glutamine, indicating that the progression of DM may disrupt energy metabolism, neurotransmission, and lipid membrane metabolism [58]. This may be related to the occurrence of CI in diabetes, so the detection of these markers may be a method for early detection of CI.

### 5.3. Brain Function Changes

Functional connectivity (FC) can be used to reflect the functional status of the brain. Studies have shown that FC topology and connectivity strength may be altered by pathological disruption [59,60,61]. FC of the brain, derived from resting-state fMRI(rs-fMRI), has gained popularity as a method for diagnosing several neurodegenerative disorders, such as AD and MCI [62]. Using rs-fMRI data, one can deduce the FC among brain regions that are structurally distinct. In this context, FC refers to the temporal correlation of the blood oxygenation level-dependent (BOLD) signal recorded from various areas of the brain [63]. The BOLD signal reflects low-frequency spontaneous fluctuations in the resting brain, which are related to intrinsic neural activity within the brain [64]. A study examining whole-brain FC using rs-fMRI found that the thalamus, angular nucleus, caudate nucleus, and paracentral lobular were highly discriminating in distinguishing patients with T2DM and MCI, patients with T2DM but no CI, and normal controls [65]. The rs-fMRI can not only use the amplitude of low-frequency fluctuations (ALFF) of BOLD signal to study the changes in brain activity related to T2DM, but also use regional homogeneity (ReHo) to study the changes in local energy consumption [66,67]. The rs-fMRI was used to detect the function of hippocampal subregions in T2DM patients. The results showed that in the T2DM group, the ALFF values for the left hippocampal CA3, subiculum, and the bilateral hippocampus amygdala transition area (HATA) were found to be elevated compared to those in HC. Additionally, the ReHo values of CA3, dentate gyrus (DG), subiculum, and HATA within the left hippocampus of individuals with T2DM were also higher than those observed in HC [68].

Traditionally, FC has been considered to be temporally quiescent, ignoring neural activity or interactions that may occur in the brain during the duration of the scan [69]. The FC in this case is usually estimated based on the length of the entire BOLD time series. It has previously been shown that the brain changes dynamically during MRI scans. FC continued to evolve even during brief scans [70]. Therefore, more and more studies have begun to focus on dynamic functional studies [70,71,72]. So far, the sliding window method has been the most frequently employed technique for analyzing dynamics in resting-state FC [72,73]. In this approach, the whole BOLD signal is divided into multiple overlapping segments, and on each segment, FC is calculated to measure the functional relationship between different brain regions at a specific time period [62]. A previous study investigated the abnormal dynamic FC of the brain in individuals with T2DM, which showed that T2DM patients had FC alterations in three key cognitive related networks (default mode network, task-positive network, and salience network [74]. Therefore, the dynamic FC may be a marker for detecting CI in diabetes mellitus.

In addition to fMRI, electroencephalogram (EEG) imaging is also an important brain functional imaging technology. EEG imaging is an inexpensive and non-invasive method. One study suggested that the decrease in absolute alpha power observed in the EEG of diabetic patients may be related to central nervous system damage in diabetes [75]. For EEG signals, phase synchronization can also be used to measure FC between groups of neurons. The phase lag index (PLI) is a FC index based on phase synchronization. A 2022 study showed that the α-band phase lag index was significantly lower in diabetic patients with MCI compared to diabetic patients without CI, and the MoCA score was positively correlated with the α-band PLI [76]. Therefore, we should also pay attention to the role of EEG imaging in identifying diabetes-related cognitive impairment.

In Table 3, we have summarized the content of the “Brain Imaging” part.

## 6. Peripheral Blood Biomarkers

### 6.1. Biomarkers Related to Neurodegeneration

The main neuropathophysiological characteristics of AD were identified as extracellular neurotic plaques of amyloid β-protein (Aβ) and intracellular neurofibrillary tangles of hyper-phosphorylated tau [77]. Tau is a microtubule-associated protein that is mainly expressed in the axons of neurons. Aβ is a peptide which is derived from a larger molecule called the amyloid precursor protein (APP). The dysregulation of insulin signaling affects APP metabolism and function, which leads to the accumulation of Aβ in the cell [78]. Studies have shown that insulin and IGF-1 can modulate tau phosphorylation by inhibiting Glycogen synthase kinase-3 beta (GSK-3β) in cultural neurons. Patients with T2DM often have insulin resistance, hyperinsulinemia, and impaired insulin signaling. Therefore, GSK-3β activity is increased in T2DM, which may lead to increased Aβ production and tau phosphorylation [78]. In the study conducted by Moran et al., patients diagnosed with T2DM exhibited markedly higher total Tau and pTau levels in the CSF [79]. These markers might offer outstanding clues for the development of diabetes-associated CI and AD.

Glycogen synthase kinase 3 (GSK3) is a serine/threonine kinase involved in various cellular processes, such as glycogen metabolism, insulin signaling, neuronal function, and so on. It was reported that higher peripheral circulating GSK-3β was negatively correlated with cognitive scores in diabetes patients diagnosed with MCI, and it has been proposed as a diagnostic marker for MCI in T2DM patients [80].

Phosphatidylinositol 3-kinase (PI3K) has been shown to be a key molecule in glucose homeostasis, closely related to the cellular uptake and utilization of glucose, and its dysfunction may be related to the increase in glucose serum levels [81]. Moreover, a deficiency of PI3K is associated with an augmentation in AD pathology and hyperphosphorylation as well as tau protein deposition through an increase in GSK-3 activity [82]. Pharmacologic treatment that increases PI3K activity has been shown to restore normal tau phosphorylation and improve cognitive dysfunction in *mice* and *rats* affected by diabetes [83,84]. Hence, reduced PI3K activity might be a potential predictor for neurodegenerative disorders in DM.

### 6.2. Markers of Inflammation

Inflammation plays an important role in the development of diabetes-related cognitive impairment. It has been reported that there is an association between inflammation and the accelerated decline in cognitive function among diabetes patients [85]. Marioni et al. explored circulating inflammatory markers (C-reactive protein (CRP), Interleukin-6 (IL-6), and Tumor Necrosis Factor-alpha (TNF-α)) in 1066 patients with T2DM and found that higher IL-6 and TNF-α levels were associated with worse cognitive scores after adjustment for sex and age [86]. A recently published meta-analysis of 7483 T2DM patients from 32 studies found moderate inverse associations between systemic IL-6, CRP, and TNF-α levels and MoCA scores, and moderate positive associations between systemic CRP and TNF-α levels and HbA1c (Hemoglobin A1c). IL-6, CRP and TNF-α are expected to be potential inflammatory biomarkers for T2DM-CI [87]. However, there was also a study involving 37 patients with T2DM that found no association between cognitive deterioration and the inflammatory markers CRP, IL-6, and TNF-α [88].

### 6.3. Advanced Glycation End Products

Advanced glycation end products (AGEs) are a set of chemicals or molecules that come into being through the non-enzymatic glycation of proteins, lipids, and nucleic acids. AGEs interact with their receptor (RAGE) and activate multiple signaling mechanisms, causing damage to different organs during the progression of diabetes. Gorska-Ciebiada et al. found that serum levels of RAGE and AGEs were significantly increased in MCI patients, which were positively correlated with HbA1c levels and negatively correlated with MoCA scores [89]. A recent systematic review and meta-analysis of 44 studies found that AGEs were elevated in T2DM patients with CI compared with those without CI [90]. A previous cross-sectional study showed that elevated serum AGE levels were associated with higher risk for MCI in patients with T2DM and were inversely associated with global cognitive function in these individuals. However, diabetic patients with MCI showed reduced serum RAGE concentration compared with diabetic patients without MCI, and the degree of reduction in serum RAGE concentration correlated with reduced executive function [91]. Given the inconsistencies among the above research findings, additional studies might be necessary to validate the potential of serum AGE and RAGE levels as biomarkers for predicting CI in diabetes.

### 6.4. Other Markers

In addition to the above markers, many studies also focus on a variety of other markers, such as miRNA. MicroRNA (miRNA) is a kind of endogenous non-coding RNA with a length of about 22 nucleotides. It plays an important role in the post-transcriptional regulation of gene expression by binding to target mRNAs. Recently, miRNAs have been proposed as potential biomarkers for the diagnosis of AD [92,93]. A study involving 163 T2DM patients showed that a significant overexpression of miR-132 was detected in T2DM patients with MCI compared to cognitively normal T2DM patients with sensitivity and specificity < 75%. Other studied miRNAs (miR-128, miR-874, miR-134, miR-323 and miR-382) had low sensitivity and specificity for detecting MCI in T2DM patients [94]. A recent study found that silent information regulator 1 combined with miR-34a-5p may be a promising biomarker for the early detection of T2DM-CI patients [95]. Brain-derived neurotrophic factor (BDNF) plays a beneficial role in the survival, growth, and proliferation of central nervous cells. It has been shown that plasma BDNF levels are lower in patients with T2DM than in those without diabetes and that plasma BDNF is inversely correlated with fasting blood glucose levels [96]. In addition, lower serum BDNF levels are associated with poor cognitive performance in T2DM patients [97]. There are also many markers, such as adipokines, islet amyloid peptide, and glycosylated hemoglobin, which have been verified by many studies as potential early identification markers of diabetes-related cognitive impairment.

In order to understand the above markers more clearly and intuitively, we have summarized them in a table (as shown in Table 4).

## 7. Discussion and Conclusions

**Figure 1:**
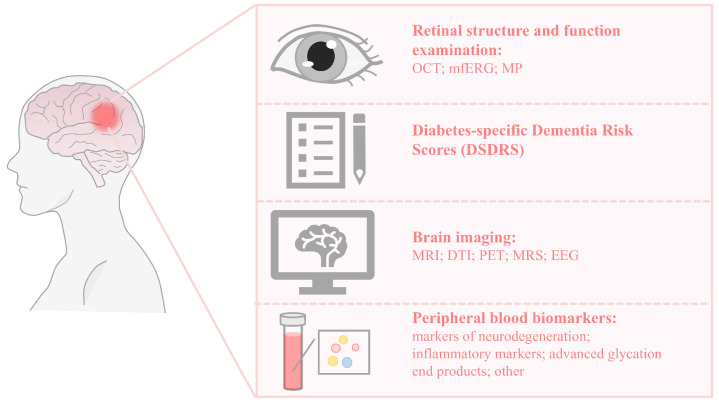
Identification and diagnostic methods for cognitive impairment in type 2 diabetes.

T2DM is associated with CI and dementia. The early detection of persons with CI is important because this group is at higher risk for difficulties with diabetes self-management. However, there is still a lack of reliable biomarkers in clinical practice, which makes the early prediction and treatment of T2DM-CI difficult. Therefore, this review summarizes the new tools for the early diagnosis of T2DM-CI that have been studied in recent years (as shown in Figure 1 and Table 5). The current ADA guidelines still recommend MMSE and MoCA as screening tests, but their sensitivity and specificity for identifying MCI are relatively low. Building on the mechanisms by which diabetes leads to CI and the pathological links between diabetes and various neurodegenerative diseases, such as AD, researchers have investigated the potential of different biomarkers as tools for early detection. These include retinal biomarkers, brain imaging biomarkers, and peripheral blood biomarkers, as discussed in this article. At the same time, some researchers have focused on the patients’ basic information and clinical symptoms to develop a tool for assessing the risk of CI in diabetic patients, known as the DSDRS (diabetes-specific dementia risk score). However, there is ongoing debate regarding the accuracy of these methods and biomarkers for detecting CI in diabetes, with inconsistent results across studies. Therefore, further large-scale research is needed to validate these diagnostic approaches.

## Figures and Tables

**Table 1:** Differences between MMSE and MoCA.

	Advantage	Deficiency	Application Situation
MMSE	Fast administration; wide international translation and use; high acceptance among professionals and non-professionals	The recognition sensitivity of patients with MCI was low; influenced by age, education, race and other factors	Moderate-to-severe cognitive impairment
MoCA	More effective than MMSE in identifying MCI	Scores are influenced by education and age	MCI; dementia

MMSE, Mini-Mental State Examination; MoCA, Montreal Cognitive Assessment; MCI, mild cognitive impairment.

**Table 2:** Retinal detection methods for diabetes-associated cognitive impairment.

	Content	Main Indicators	Application	Limitations
Optical coherence tomography (OCT)	Measure the thickness of the retinal nerve fiber layer (RNFL) and other retinal layers	RNFL thickness; retinal layer thickness	Used for early detection of retinal diseases, RNFL thinning is associated with cognitive decline and may serve as a screening tool for cognitive decline	Lack of universally accepted cutoffs; potential variability; focus on structural changes
Multifocal electroretinogram (mfERG)	Record the electrical activity of multiple retinal areas to assess retinal function	Retinal electrophysiological responses; local retinal function	Used to detect retinal neurodegenerative changes in diabetic retinopathy, which may be associated with the occurrence of diabetes-related cognitive impairment	Complex; requires specialized personnel; time-consuming
Microperimetry (MP)	Assess retinal sensitivity (photoreception) and fixation	Retinal sensitivity; ocular fixation	Can be used to assess retinal function, with decreased retinal sensitivity potentially associated with cognitive decline, showing potential as a screening tool for cognitive impairment	Device availability is limited in many healthcare settings; no studies on psychological/mood impact on the test; advanced DR may limit tool effectiveness

**Table 3:** Brain imaging techniques for detecting cognitive impairment in diabetes.

	Content of Assessment	Detection Method	Advantages	Limitations
Brain structural imaging	Changes in gray matter (GM) and white matter (WM)	VBM; SBM; DTI	Non-invasive; quantitative, automated analysis	Highly affected by motion artifacts, unable to provide functional information
Brain metabolic imaging	Changes in glucose metabolism and brain metabolites (such as NAA, Cr, and Cho)	PET;^1^H MRS	Non-invasive; capable of detecting metabolic changes at an early stage	Expensive equipment; time-consuming
Brain functional imaging	Functional connectivity (FC); spatiotemporal dynamics of brain networks	fMRI; EEG	Non-invasive; dynamic assessment of brain function	Requires complex calculations

VBM, voxel-based morphometry; SBM, surface-based morphometry; DTI, diffusion tensor imaging; PET, positron emission tomography; ^1^H MRS, proton magnetic resonance spectroscopy; NAA, N-acetylaspartate; Cr, creatine; Cho, choline; EEG, electroencephalogram; fMRI, functional magnetic resonance imaging.

**Table 4:** Peripheral blood markers for cognitive impairment in diabetes.

	Main Biomarkers	Relation to Diabetes	Impact On Cognitive Function	Application Potential
Neurodegenerative biomarkers	Aβ; phosphorylated Tau protein; GSK3; PI3K	Tau and Aβ accumulation, and dysfunction of PI3K and GSK-3β are related to diabetes	Abnormal levels of Tau, Aβ, PI3K, and GSK-3β are associated with cognitive decline	These biomarkers are significantly associated with diabetes-related cognitive impairment, including MCI and Alzheimer’s disease
Inflammatory markers	CRP; IL-6; TNF-α	High inflammation levels are related to insulin resistance and glucose metabolism disorders in diabetes	Elevated inflammatory markers (such as CRP, IL-6, TNF-α) are associated with cognitive decline	CRP, IL-6, and TNF-α may serve as biomarkers for diabetes-related cognitive impairment, but research findings are inconsistent
Advanced glycation end products	AGEs; RAGE	AGEs and RAGE levels are elevated in diabetic patients, especially under poor blood sugar control	High levels of AGEs are associated with cognitive dysfunction	AGEs and RAGE may serve as biomarkers for diabetes-related cognitive impairment, but further research is needed to confirm their potential
Other biomarkers	miRNA; BDNF; adipokines; islet amyloid peptide; glycosylated hemoglobin			

Aβ, amyloid β-protein; GSK3, glycogen synthase kinase 3; PI3K, phosphatidylinositol 3-kinase; CRP, C-reactive protein; IL-6, interleukin-6; TNF-α, tumor necrosis factor-α; AGEs, advanced glycation end products; RAGE, receptor for advanced glycation end products; miRNA, microRNA; BDNF, brain-derived neurotrophic factor. The blank represents no content, as the information is too extensive and diverse to be unified.

**Table 5:** Summary of the four diagnostic methods.

	Content	Main Indicators/Biomarkers	Application
Retinal Structure and Function Examination	OCT	RNFL thickness; retinal layer thickness	Used for the early detection of retinal diseases, RNFL thinning is associated with cognitive decline and may serve as a screening tool for cognitive decline
	mfERG	Retinal electrophysiological responses; local retinal function	Used to detect retinal neurodegenerative changes in diabetic retinopathy, which may be associated with the occurrence of diabetes-related cognitive impairment
	MP	Retinal sensitivity; ocular fixation	Can be used to assess retinal function, with decreased retinal sensitivity potentially associated with cognitive decline, showing potential as a screening tool for cognitive impairment
Diabetes-Specific Dementia Risk Score	DSDRS	Score	Estimates a person’s overall risk of developing dementia within the next decade based on diabetes-related co-morbidities and complications, age, and education level
Brain Imaging	Brain Structural Changes	Changes in gray matter (GM) and white matter (WM)	Enabling the early detection of brain structural abnormalities in T2DM patients and serving as a structural basis for screening diabetes-related cognitive impairment
	Brain metabolic imaging	Changes in glucose metabolism and brain metabolites (such as NAA, Cr, and Cho)	Providing metabolic biomarkers for the early screening of cognitive decline
	Brain functional imaging	Functional connectivity (FC); spatiotemporal dynamics of brain networks	Revealing abnormal brain network activities in T2DM patients and offering functional biomarkers for screening cognitive dysfunction
Peripheral Blood Biomarkers	Neurodegenerative biomarkers	Aβ; phosphorylated Tau protein; GSK3; PI3K	These biomarkers are significantly associated with diabetes-related cognitive impairment, including MCI and Alzheimer’s disease
	Inflammatory markers	CRP; IL-6; TNF-α	CRP, IL-6, and TNF-α may serve as biomarkers for diabetes-related cognitive impairment, but research findings are inconsistent
	Advanced glycation end products	AGEs; RAGE	AGEs and RAGE may serve as biomarkers for diabetes-related cognitive impairment, but further research is needed to confirm their potential
	Other biomarkers	miRNA; BDNF; adipokines; islet amyloid peptide; glycosylated hemoglobin	
